# Cardiac Ultrasound in the Intensive Care Unit: A Review

**DOI:** 10.7759/cureus.4612

**Published:** 2019-05-07

**Authors:** Avani R Patel, Amar R Patel, Shivank Singh, Shantanu Singh, Imran Khawaja

**Affiliations:** 1 Internal Medicine, Northern California Kaiser Permanente, Fremont, USA; 2 Internal Medicine, Southern Medical University, Guangzhou, CHN; 3 Pulmonary Medicine, Marshall University School of Medicine, Huntington, USA

**Keywords:** transthoracic echocardiography, ultrasound, hemodynamic echocardiography, focused ultrasound, limited transthoracic echocardiogram, focused assessment with transthoracic echocardiography, bedside echocardiographic assessment in trauma/critical care, rapid ultrasound for shock and hypotension, focused rapid echocardiographic evaluation, transducer

## Abstract

Transthoracic echocardiography (TTE) is an incredibly valuable tool in today’s emergency rooms and intensive care units (ICUs). It has the ability to provide a complete evaluation of the structure and function of the heart, the valves, stroke volume, ejection fraction, and much more. Previous academic studies have also determined that point-of-care TTE done by non-cardiologist physicians can also provide higher accuracy in patient assessment and management, with potential prognostic impact by assessing the severity of cardiac dysfunction and response to treatment. The aim of this review article is to examine further these point-of-care evaluations, what they entail, their benefits, and where further research would better our own understanding.

## Introduction and background

Echocardiography as a tool was first introduced in 1953 by a cardiologist named Inge Edler, working with a physicist named Carl Hertz [1]. Today, echocardiography has progressed to the point where it has become an invaluable tool for any treating physician in any department. As the medical community has become more technologically savvy, transthoracic echocardiography (TTE) has become a primary mode of assessment in both the emergency department and the intensive care unit (ICU) [2]. Research done has also determined that point-of-care TTE provides higher accuracy in patient assessment and management, with a potential prognostic impact by assessing the severity of cardiac dysfunction and response to treatment [2]. In 2010, a combined statement between the American Society of Echo and the American College of Emergency Physicians was issued on the subject of point-of-care cardiac evaluations being performed by non-cardiologists [3]. In that statement, it was decided that the term focused cardiac ultrasound (FOCUS) should be used to describe point-of-care evaluations performed by non-cardiologists, thus allowing differentiation from a comprehensive TTE and a limited echo, which are exclusively performed by cardiologists [3]. Later on, in 2013, the American Society of Echo decided that FOCUS should be used as an adjunct to the physical exam in an emergency setting, off hours, or when formal echo is not available [3]. These guidelines further state that FOCUS should be used specifically for the evaluation of cardiac tamponade, effusion, global heart function, and intravascular volume [3]. There should also be an intent to follow up with a formal echo for the confirmation of findings from the FOCUS exam [3]. The aim of the following review article is to better understand the use of cardiac ultrasound in the intensive care unit (ICU), the different views used, and the relevant exams, and to address the limits of our current understanding. In doing so, future endeavors can be made to address those gaps.

## Review

Different views

**Figure 1:**
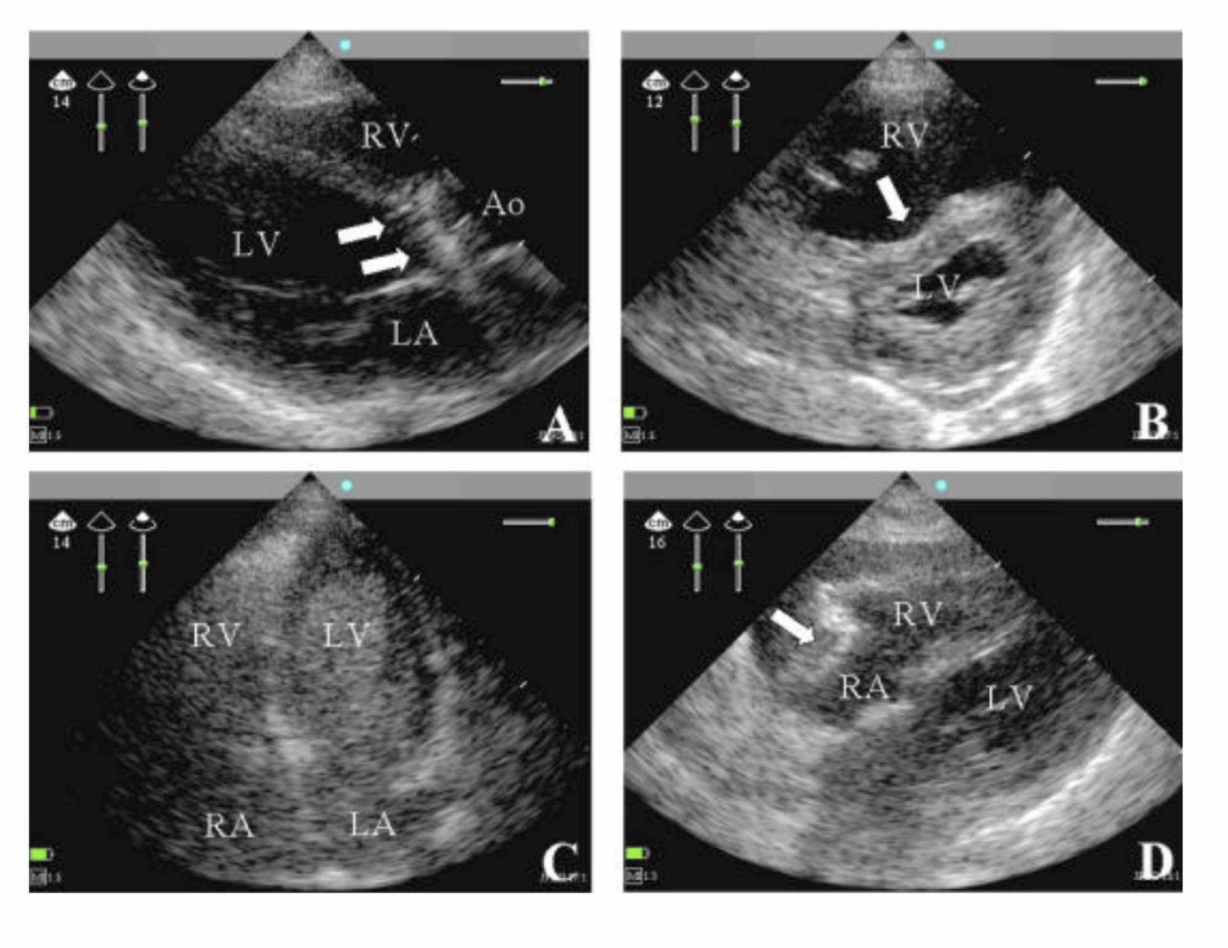
(Panels A-D) Views from Four Different Patients (Panel A) The parasternal long-axis (PLA) view was taken from a patient with septic shock. The patient had severe aortic endocarditis (arrows pointing to vegetations present on the aortic valve) and a dilated left ventricle. The aorta (Ao), the right ventricle (RV), and the left atrium (LA) are also seen [6]. (Panel B) The parasternal short-axis (SA) view was taken from a patient with acute respiratory distress syndrome and associated right heart dysfunction. The right ventricle (RV) is shown to be enlarged due to severe pulmonary hypertension (seen with arrow) [6]. (Panel C) The apical (AP) view was taken from a ventilated patient with refractory hypoxemia. The right ventricle (RV), the left ventricle (LV), the right atrium (RA), and the left atrium (LA) all are seen [6]. (Panel D) The subxiphoid (SX) view was taken from a patient with shock and pulsus paradoxus. Pericardial effusion is present (pointed out by the arrow). Also seen are the right ventricle (RV), the right atrium (RA), and the left ventricle (LV) [6].

Although the objective of FOCUS exams versus comprehensive transthoracic echocardiography (TTE) is to perform a very standardized evaluation as compared to a comprehensive one, the technical approach is essentially unchanged [3]. Four views are taken. They are the parasternal long axis (PLA) view, the parasternal short axis (SA) view, the apical (AP) view, and the subxiphoid (SX) view (see Figure 1). Both the PLA and SA views can quantify the cardiac ejection fraction and both can be taken in more than 90% of patients [4-5]. It is necessary to use a phased array or a small footprint probe in order to look "between" the ribs. If that is not available, then it is still possible to do the full evaluation of basic cardiac function with any low-frequency transducer.

The Parasternal Long-axis (PLA) View

In order to take the PLA view, the patient is put in the supine or in the slightly left lateral position. The transducer or probe is placed on the chest, slightly left of the sternum at the third or fourth intercostal space [3]. The transducer is oriented towards the right mid-clavicular line and can be moved up or down, with the final goal of dividing the left ventricle on its longitudinal axis [3]. With the PLA view, a physician can observe the left ventricle, the cross-sections of the mitral and aortic valve, and part of the right ventricle. With the PLA view, physicians can evaluate left ventricle (LV) contractility, pericardial effusions, and pleural effusions.

*The Parasternal Short-axis (SA)* *View*

The next view taken after the PLA view is the SA view. The SA view is taken by rotating the probe 90-degrees clockwise to bisect the left mid-clavicular line in the fourth intercostal space [3]. The SA view will show a cross-sectional view across the left ventricle. Therefore, the aortic valve, the mitral valve, the papillary muscles, and the apex of the heart can be visualized with the SA view. The SA view is considered the best for evaluating right heart function.

The Apical (AP) View

The AP view is found by placing the probe near the apex of the heart. This is usually a few centimeters below the nipple and between the left mid-clavicular line and anterior axillary line [3]. Turning the patient onto the left lateral position can sometimes bring the heart closer to the chest wall, making it easier to visualize. The AP view shows a view of the heart that is divided in an anterior-posterior orientation. It shows the right atrium (RA), the right ventricle (RV), the left atrium (LA), and the left ventricle (LV). The real value of the AP view is that with some forward manipulation of the probe, it can also visualize the left ventricular outflow tract to the aorta, therefore, giving a five-chamber view. The AP view is helpful in assessing for aortic stenosis, stroke volume, cardiac index, ejection fraction (EF), right heart function, and heart size. Usually, the RV is about 60% of the LV [4]. The ratio is higher in cases of right-sided heart failure, right-sided heart overload, or in mechanically ventilated patients.

*The Subxiphoid (SX)* *View*

Unlike the others, the subxiphoid (SX) view is taken from the abdomen, looking across the left lobe of the liver up towards the heart [3]. Across the liver, the inferior vena cava (IVC) can be seen and followed up to where it joins the right side of the heart. The SX view is valued for evaluating cardiac activity and pericardial effusion. It is also valued because it is the only view that can be taken in mechanically ventilated patients with high mean airway pressure settings. It is also the typical view taken in the focused assessment sonogram for trauma (FAST). FAST is an ultrasound protocol developed to assess for hemoperitoneum and hemopericardium [7]. Several studies have demonstrated sensitivities between 85% to 96% and specificities exceeding 98% [8]. Experienced providers perform the FAST exam in less than five minutes [9], and its use decreases time to surgical intervention, patient length of stay, and rates of computed tomography and diagnostic peritoneal lavage (DPL) [10].

Different types of FOCUS exams

A FOCUS exam is a bedside ultrasound examination of the heart performed by a non-cardiologist physician as an adjunct to their physical examination [11]. The goal of a FOCUS exam is to recognize a narrow list of abnormalities that are both detectable by physicians with limited ultrasound training and have high clinical assessment value [11]. Previous research studies have come up with different types of FOCUS exams. Despite the fact that more studies need to be done to validate FOCUS exam utilization, it is apparent that they may improve clinical outcomes [12].

Bedside Echocardiographic Assessment in Trauma/Critical Care

Bedside echocardiographic assessment in trauma/critical care (BEAT) was a point-of-care cardiac exam developed in 2008 by a group of surgeons. BEAT was developed in response to the idea that pulmonary artery (PA) catheter-guided resuscitation may not be of benefit [13]. As its name suggests, BEAT is utilized in the trauma setting. It utilizes all four of the same views: PLA, SA, AP, and SX. It evaluates volume status by mitral inflow and IVC diameter and collapsibility. It goes on to further evaluate for pericardial effusion, LV function, and, lastly, RV function and dilation [12].

Focused Assessment with Transthoracic Echocardiography

The focused assessment with transthoracic echocardiography (FATE) is a FOCUS exam that was created for general critical care. It utilizes the same four standard views as TTE: the PLA, SA, SX, and AP views. FATE evaluates for pericardial effusion, LV function, RV function and/or dilation, and lungs [14].

Rapid Ultrasound for Shock and Hypotension

In 2013, the rapid ultrasound for shock and hypotension (RUSH) exam was created. It later became increasingly popular in the emergency medicine community. Its objective is to assess for sources of hypotension in the undifferentiated shock patient [15]. Like other FOCUS exams, there are four views utilized: the PLA, SA, SX, and AP views. They evaluate left and right heart size and function, effusion, and IVC size and collapse. With the SA view, if the measured size difference between systole and diastole is less than 30%, this will indicate abnormal function [3]. Unlike other FOCUS exams, the RUSH exam also evaluates the lungs, aorta, and leg veins for deep vein thrombosis (DVT) [15].

Limited Transthoracic Echocardiogram

In 2014, Dr. Paula Ferrada came up with the limited transthoracic echocardiogram (LTTE) and the ABCD (Asynergy, B-lines, Contractile reserve, Doppler flowmetry) echo, a tool for the initial assessment of hypotensive patients in the trauma bay. Her work describes a simplified exam that evaluates cardiac function (good vs. poor), volume status (IVC fat vs. flat), and pericardial effusion (present or absent) [16]. The randomized clinical trial performed had 215 patients, with 92 in the LTTE group and 123 in the non-LTTE group. It was shown that therapy was modified in 41% of the LTTE group patients and in 96% of patients older than 65 years. In the follow-up, LTTE-guided resuscitation a showed statistically shorter time to diagnosis, time to the operating room, higher ICU admissions, and lower mortality than their non-LTTE group counterparts [16].

Hemodynamic echocardiography

For many, hemodynamic echocardiography is simply the next step for directing care in resuscitative management, especially in busy emergency rooms and ICUs. The FOCUS exams described previously are designed to provide an evaluation of cardiac function and volume status with any available machine. In comparison, hemodynamic echocardiography requires a full-service cardiovascular machine capable of accurate Doppler measurements. The reason it is so important is because the next question asked after determining volume status in an intensive care setting would be whether fluid or inotropic support is better in a given individual [3]. A 21-year old with hypovolemic shock would need blood and possibly a vasopressor. An 80-year pneumonia patient might need fluids, inotropic support, plus a vasoconstrictor [3]. More data needs to be known before proceeding further, which is where hemodynamic echography would be most beneficial.

Focused Rapid Echocardiographic Evaluation

Focused rapid echocardiographic evaluation (FREE) is one of the best-described types of hemodynamic echocardiography [5,17]. Compared to a TTE, the FREE exam is functionally evaluated rather than anatomically [3]. FREE evaluates cardiac function, volume status, vascular resistance, and anatomy. FREE evaluates these by utilizing the patient’s mean arterial blood pressure and body surface area. Then by using the measurements and evaluations, patients are grouped into one of four hemodynamic profiles. The profiles are normal x 3, vasodilated/high output, hummingbird, and dysfunctional. Using this information, if the patient is in shock, then the type of shock can also be determined.

Normal x 3 heart is a hemodynamic profile that describes a normal EF and a normal cardiac output. The patient will appear volume responsive (to intravascular fluid replacement) with normal peripheral resistance. From that and the patient’s history, if end-organ-hypoperfusion is the concern, then fluid replacement would be the next natural step. However, if the patient history is supportive of respiratory failure, then diuresis would be the next natural step of management.

A vasodilated/high-output hemodynamic profile refers to a patient with normal cardiac function and decreased systemic vascular resistance, either secondary to diffuse arteriolar dilation or possible bypass of the arterioles and capillary beds, leading to activation of neurohormones [18-19]. If a patient has this profile, then vasoconstrictors need to be started, but depending on the patient volume status, fluids may or may not be started with the vasoconstrictors.

The hummingbird hemodynamic profile indicates that the patient’s heart is under-filled, leading to a low stroke volume and high heart rate. This combination of low stroke volume and high heart rate will put the patient at risk for myocardial stress, especially if the patient has any significant cardiovascular history. Therefore, in those profiles, regardless of whether there is underlying end-organ-hypoperfusion or respiratory failure, the recommended next step is fluid replacement.

The fourth and final hemodynamic profile is the dysfunctional hemodynamic profile. The profile is as stated. Typically, these patients need to be managed with a great deal of care and cannot receive as much fluid replacement. Therefore, the recommended next step is inotropic support and the judicious management of fluid replacement [3].

## Conclusions

The material reviewed in this paper focuses on the use of cardiac ultrasound in the ICU, the different views used, the relevant exams, and to address the limits of our current understanding. It goes into detail regarding the use of FOCUS exams for targeted cardiac evaluations and their benefits. Despite these key points being addressed, larger studies are needed to better understand FOCUS exams and hemodynamic echocardiography exams. Only with more data can we adjust and create more useful protocols regarding these exams and be assured that the patient will still receive the best level of care. This is a review article for busy physicians to have a cumulative view of our current situation regarding the use of echocardiography and FOCUS exams.
